# Evaluation of biofilm colonization on multi-part dental implants in a rat model

**DOI:** 10.1186/s12903-021-01665-2

**Published:** 2021-06-18

**Authors:** Eva Blank, Jasmin Grischke, Andreas Winkel, Joerg Eberhard, Nadine Kommerein, Katharina Doll, Ines Yang, Meike Stiesch

**Affiliations:** 1 Department of Dental Prosthetics and Biomedical Materials Science, Hannover Medical School, Carl-Neuberg-Str. 1, 30625 Hannover, Germany; 2 The University of Sydney Dental School & The Charles Perkins Centre, Faculty of Medicine and Health, The University of Sydney, Sydney, NSW Australia; 3 Lower Saxony Centre for Biomedical Engineering, Implant Research and Development (NIFE), Stadtfelddamm 34, 30625 Hannover, Germany

**Keywords:** Animal model, Dental implant, Titanium implant, Peri-implant mucositis, Implant-associated infection, Biofilm

## Abstract

**Background:**

Peri-implant mucositis and peri-implantitis are highly prevalent biofilm-associated diseases affecting the tissues surrounding dental implants. As antibiotic treatment is ineffective to fully cure biofilm mediated infections, antimicrobial modifications of implants to reduce or prevent bacterial colonization are called for. Preclinical in vivo evaluation of the functionality of new or modified implant materials concerning bacterial colonization and peri-implant health is needed to allow progress in this research field. For this purpose reliable animal models are needed.

**Methods:**

Custom made endosseous dental implants were installed in female Sprague Dawley rats following a newly established three-step implantation procedure. After healing of the bone and soft tissue, the animals were assigned to two groups. Group A received a continuous antibiotic treatment for 7 weeks, while group B was repeatedly orally inoculated with human-derived strains of *Streptococcus oralis*, *Fusobacterium nucleatum* and *Porphyromonas gingivalis* for six weeks, followed by 1 week without inoculation. At the end of the experiment, implantation sites were clinically assessed and biofilm colonization was quantified via confocal laser scanning microscopy. Biofilm samples were tested for presence of the administered bacteria via PCR analysis.

**Results:**

The inner part of the custom made implant screw could be identified as a site of reliable biofilm formation in vivo*. S. oralis* and *F. nucleatum* were detectable only in the biofilm samples from group B animals. *P. gingivalis* was not detectable in samples from either group. Quantification of the biofilm volume on the implant material revealed no statistically significant differences between the treatment groups. Clinical inspection of implants in group B animals showed signs of mild to moderate peri-implant mucositis (4 out of 6) whereas the mucosa of group A animals appeared healthy (8/8). The difference in the mucosa health status between the treatment groups was statistically significant (*p* = 0.015).

**Conclusions:**

We developed a new rodent model for the preclinical evaluation of dental implant materials with a special focus on the early biofilm colonization including human-derived oral bacteria. Reliable biofilm quantification on the implant surface and the symptoms of peri-implant mucositis of the bacterially inoculated animals will serve as a readout for experimental evaluation of biofilm-reducing modifications of implant materials.

## Background

Implants are of increasing importance in several medical disciplines, which is due to both technical advances and growing demand in ageing populations. Although endosseus dental implants increase the life quality of affected patients [1, 2], implant sites are prone to bacterial infections [3, 4]. A particular threat to the health of implant-surrounding tissues are pathogenic bacterial biofilms located on the implant surface [5, 6]. Without adequate treatment, accumulation of bacterial biofilms on dental implant surfaces can lead to peri-implant mucositis, an inflammation of the peri-implant soft tissue which affects more than 50% of dental implants [7–9]. A randomized controlled trial found management of peri-implant mucositis to be successful in only 70% of implants after 12 weeks [10]. Without resolution mucositis may progress to peri-implantitis, which also affects the underlaying alveolar bone and occurs at more than 25% of implants after 5 years [9]. Ensuing sequelae may include bone regression and ultimately implant loss [11–13].

Biofilms develop on teeth and dental implants alike [14]. Biofilm colonization typically follows a certain progression of organisms being incorporated into the biofilm, with specific genera fulfilling key functions in this process. Oral biofilm organisms can be classified as early and late colonizers [15]. Early colonization of teeth and titanium implants is dominated by streptococci species like *Streptococcus sanguinis*, *Streptococcus mitis* and *Streptococcus oralis* (*S. oralis*) and actinomyces species [16–19]. These pioneer organisms, which coaggregate extensively with each other, are needed to attach to the salivary pellicle and establish a kind of substrate for further bacterial attachment [20–22]. Among the late colonizers there are several species, which strongly correlate with periodontal disease, like the “red complex” cluster of *Porphyromonas gingivalis (P. gingivalis), Treponema denticola* and *Tannerella forsythia*. The integration of these species in the biofilm actually depends on the presence of *Fusobacterium nucleatum *(*F. nucleatum*) and other bacteria of the “orange complex” [23–25]. *F. nucleatum* is one of the most frequently found species in dental plaque [26] and is known to function as a “bridge organism” between early and late colonizers due to its ability to coaggregate with a variety of other species [15, 27].

The bacterial composition of implant-associated biofilms is highly predictive for the clinical state of the implant. Interestingly, while there are several species involved in both conditions, the microbial profile of periodontal disease and peri-implant infections are not completely identical [28]. In a very recent publication by Ghensi and colleagues, which focused on the identification of microbiota and expression profiles associated with peri-implantitis and peri-implant mucositis, *P. gingivalis* and *F. nucleatum* were both found to be strongly associated with peri-implantitis while *F. nucleatum* alone also showed a strong correlation with the preceding peri-implant mucositis [8]. This might be expected, considering its function as a key bridge organism in the development of pathogenic oral biofilms [8]. Well-established biofilms are resistant against both the patient’s immune system and antibiotic treatments [29], often leaving only mechanical removal as a treatment option [14]. Development of these infectious biofilms has to be prevented, especially at sites, which cannot be adequately reached by routine tooth brushing or flossing, like the interstices between the components of multi-part implant systems. Bacterial leakage from such sites has been reported to threaten implant health [30, 31]. To achieve a reduction of biofilm colonization new development of implant materials and anti-bacterial surface modifications is paramount.

Before new or modified implant materials can be used in patients, both intended and unintended effects have to be evaluated via preclinical testing in vitro and in suitable animal models. For the assessment of the efficacy of a new implant material, an in vivo model which mimics the initial stages of the development of peri-implant infections in a patient is desirable.

Today, mainly large animal models, like dogs and pigs [32–35], are used to test new implant designs and for studies on dental peri-implant infections. However, with regard to housing complexity and possible ethical issues, there is an increasing interest in easier-to-handle small animal models. Most existing rodent models are still under development regarding the methods for implantation and induction of pathogenic processes. In general, the focus of these models is not on the quantification of implant-associated biofilms, but on etiology, pathology and therapy of peri-implant infections. Furthermore, the applied methods do not always intend the induction of peri-implant-infections via the naturally occurring way of successive biofilm colonization of the implant surface. Current treatment procedures include ligatures tied around implants to facilitate the accumulation of bacteria, implants that are colonized with a human pathogen in vitro prior to implantation [36–38], or lipopolysaccharid injections into the implant-surrounding soft tissue to induce peri-implantitis-like symptoms [39, 40]. To our knowledge, there is currently only one model for experimental induction of peri-implantitis in rats via oral application of human-derived pathogenic bacteria [41]. However, the formation of bacterial biofilms on the implants was not assessed in this study.

For the in vivo evaluation of antibacterial implant materials, a specific setup is needed, which allows for the reliable analysis of these bacterial biofilms.

Therefore, the aim of this study was to develop a small animal model to analyze biofilm colonization on dental implants. To ensure easier handling and maintenance, and allowing for sufficiently large numbers of animals per test group, rats were used.

They also offered a limited diversity of oral microbiota to guarantee reproducible testing conditions. To mimic human implant dentistry, a multi-part titanium implant system which resembles implant models currently used in implant dentistry was inserted into the maxilla in a three-stage implantation process. As human-derived oral primary colonizers and pathogenic bacteria should be part of the biofilm colonizing the implant, and the biofilm formation should occur naturally, key species were administered orally. Using this model, reliable biofilm colonization was verified using fluorescence staining and confocal laser-scanning microscopy and the integration of the administered bacteria was assessed by strain-specific PCR. Additional, clinical signs of infection were detected and evaluated by a dentist and scored using an established method.

## Methods

The aims of this study were

1. To establish a novel three-step implantation method for the installation of a multi-part experimental implant in the rat.
2. To find out if an introduction of the human-derived oral bacteria *S. oralis*, *F. nucleatum* and *P. gingivalis*, which are known to be critical components for human oral biofilm development and pathogenic maturation, is possible in specific pathogen-free Sprague Dawley rats.
3. To establish a method for reproducible biofilm quantification as a readout for later evaluation of innovative implant materials in this in vivo setting.

### Animals

Experimental implantations were performed in 27 specific pathogen-free female ex-breeder Sprague-Dawley rats 47–48 weeks of age, which had been purchased from Charles River Laboratories (Sulzfeld, Germany). Animal numbers were chosen based on prior experience [42]. Throughout the experiment, rats were housed under SPF conditions, including nesting material, with free access to chow (1320 Maintenance diet for rats and mice, Altromin, Spezialfutter GmbH&Co.KG, Lage, Germany) and water ad libitum. The overall health status of all animals was checked daily. The implant-specific health status and the weight of the animals was documented every other day throughout the experiment, starting after the first surgery. Exclusion criteria were determined before the start of the experiment and included the following conditions: weight loss of 20% or more at any time point of the experiment, which would also have resulted in immediate euthanasia of the animal, loss of both implants, or death before conclusion of the experiment. Four animals were lost over the course of the experiment as detailed in the results section. The remaining 23 animals were included in the final analysis of this study. In order to minimize animal stress, the animals were housed in groups of three per cage until the assignment to the different treatment groups after intervention three.

### Experimental procedure

The experiment was carried out in three stages (each initiated by an intervention under anaesthesia).

**Fig. 1:**
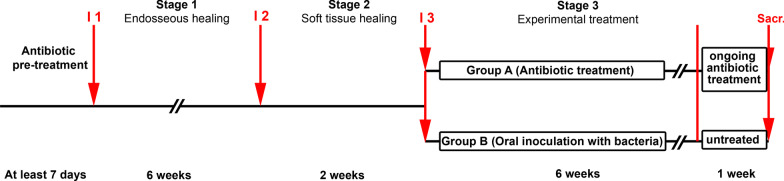
Time line of the experimental procedure of implant installation. I1, Intervention 1: Endosseous implantation; I2, Intervention 2: Placement of healing abutments; I3 = Intervention 3: Placement of experimental abutments

The experimental procedure is depicted in Fig. 1. Rats were pre-treated daily with a combination of ampicillin (Carl Roth GmbH &Co. KG, Karlsruhe, Germany) and kanamycin (Carl Roth GmbH &Co. KG, Karlsruhe, Germany), 20 mg each, dissolved in 200 µl of ultrapure-water for at least 7 days by oral application. For all animals, antibiotic treatment was continued throughout the stages 1 and 2 to prevent infection of the peri-implant tissues during the implant insertation and healing phases, as well as to suppress the endogenous bacterial flora of the rats to facilitate the colonization by human-derived species [43–45]. During the different interventions, rats were anaesthetised by intraperitoneal injection of 85 mg/kg ketamine (Anesketin; Albrechts GmbH, Aulendorf, Germany) and 4–6 mg/kg xylazine (Sedaxylan; Albrechts GmbH, Aulendorf, Germany). To protect the eyes of the rats from chafing or drying during surgery, they were covered with a generous amount of dexpanthenol eye-ointment (Bepanthen®, Bayer AG, Leverkusen, Germany).

#### Stage 1: Implantation and endosseous healing

**Fig. 2:**
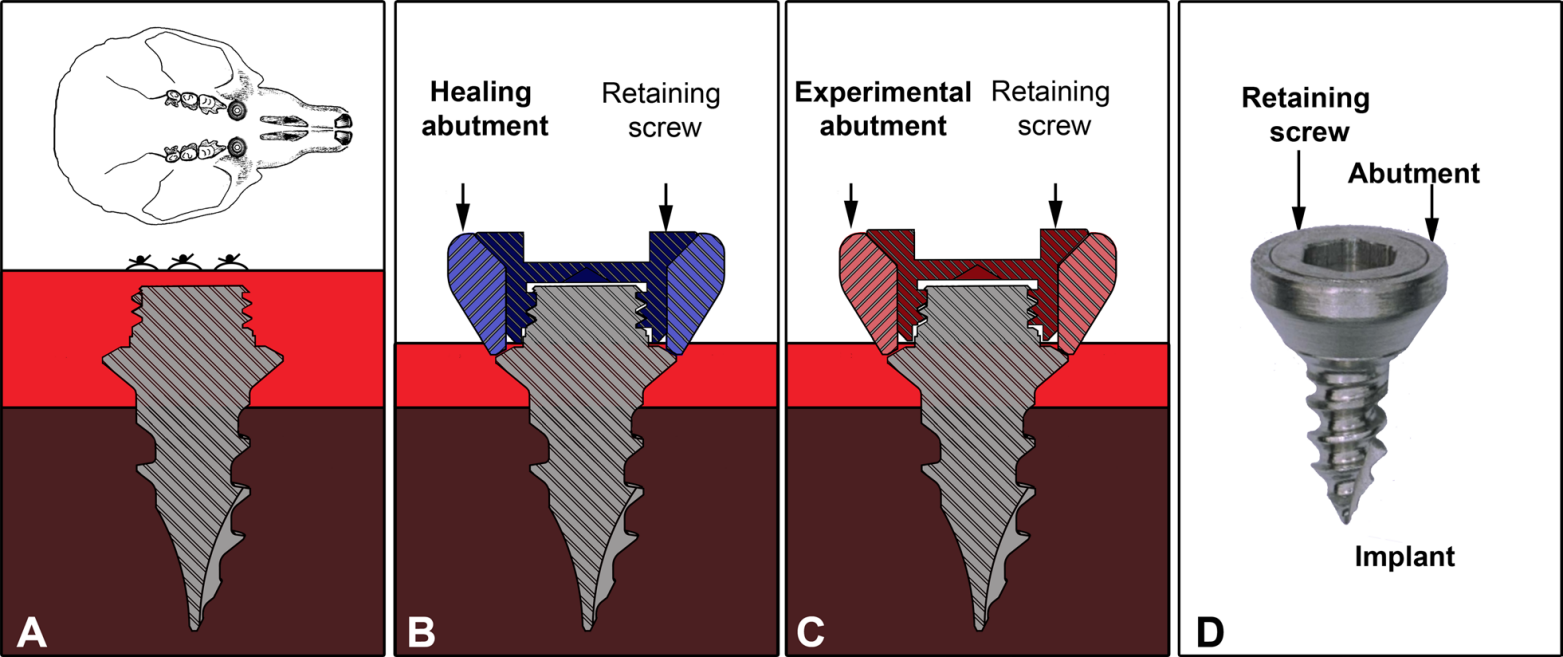
Systematic illustration of the implantation process. **A** In intervention 1 titanium implants are placed bilaterally in the diastema region of the upper jaw. Implants are screwed into the jaw bone and covered by a mucosa flap. The implantation is followed by a healing period of 6 weeks (stage 1) to allow healing of the mucosa and osseous integration. **B** In intervention 2 the mucosa covering the implant is reopened if necessary and the titanium healing abutment is attached to the implant with the help of a titanium retaining screw. Placement of the healing abutments is followed by a two-week period to allow healing of the mucosa (stage 2). **C** In intervention 3 the healing abutments are replaced by experimental abutments. Each animal is fitted with one unmodified titanium abutment and retaining screw on one side and one functionalized abutment and retaining screw on the opposite side, if possible (for details of the success rate at any step of the implantation procedure please refer to the main text). **D** Completely assembled implantation construct as it resides in the oral cavity during the final treatment period of 7 weeks

A mesio-distal incision of the gingiva of about 4 mm length was made bilaterally in the diastema region of the alveolar ridge of the maxilla, anterior to the first molar using a disposable scalpel (FEATHER SAFETY RAZOR CO., LTD, Osaka, Japan). Once the submucosal bone was exposed, a pilot burr (Gebr. Brasseler GmbH & CO. KG, Lemgo, Germany) was sunk 1.5 mm deep into the palatal bone to prepare an implant bed. Self- cutting custom-made, titanium implants (Gebr. Brasseler GmbH& CO. KG) with a diameter of 1.8 mm and a thread length of 2.4 mm were manually screwed in clockwise using an equipped screwdriver until no further movement was possible (Fig. 2A). Implants were carefully positioned in such a way that neither the buccal bone nor the sinus was perforated. The mucosa was mobilized in a minimally invasive way by creating a split flap around the implants to enable submerged initial healing. The incision was sutured with non-resorbable suture material (PremiCron 6/0, B. Braun Surgical, S.A. Rubi. Spain). The implantation was followed by a period of 6 weeks for healing and osseous integration.

#### Stage 2: Placement of healing abutments and healing of soft tissue

Six weeks after the implantation, transmucosal healing abutments were attached to the implant screws. To achieve this, implant heads were laid bare at implantation sites with fully covered implants. A small incision, about 2–3 mm in length, was made at the point where the tip of the implant’s upper thread was palpable through the mucosa. At implantation sites with only partially covered implants, the implant head was laid bare, with a minor incision, or the gingiva was mobilized using a dental probe with a blunt end if necessary. Custom-made titanium healing abutments (Gebr. Brasseler GmbH & CO. KG) were placed to generate a trans-mucosal passage at the implantation site (Fig. 2B). Healing abutments were fitted on the upper thread of the implant and attached with a retaining screw.

#### Stage 3: Placement of experimental abutments and experimental treatment

In the final intervention, healing abutments were replaced with experimental abutments (Gebr. Brasseler GmbH & CO. KG) (Fig. 2C), which remained in place during the final stage of the experiment and were used for biofilm analysis. All remaining implants were fitted with a new titanium abutment and retaining screw, structurally identical with the previous healing abutments. In animals, which still had both implants in place, one of the implants was fitted with an abutment and retaining screw combination which additionally featured a surface functionalisation that will be described elsewhere, while abutment and retaining screw on the opposite side were unmodified.

### Experimental treatment groups

Twenty-three animals that had been successfully fitted with experimental abutments in intervention 3 were there after divided into two treatment groups. Twelve animals in the control group (A) were continuously treated with ampicillin and kanamycin as described above during the following 7 weeks. For the eleven animals of the experimental group (B), antibiotic treatment was terminated the day after the third intervention. After the arbitrary assignment of the animals to treatment or control group the animals were reassigned to cages containing two animals of the same treatment group each if socially compatible. Some animals had to be kept in single cages after that time point, as no socially compatible partner was available within the same treatment group. From then on, animals of experimental group B were inoculated orally with a combination of *P. gingivalis, F. nucleatum* and *S. oralis* during the following six weeks. In the seventh week, the rats in group B did not receive any treatment. The researcher who conducted the experiment was aware of the group allocation at all times during the experiment until the dissection.

### Bacterial strains and culture conditions

The oral microbiota of the experimental group rats was supplemented with human-derived strains of three key biofilm- or peri-implantitis-associated bacterial species: *P. gingivalis* (American Type Culture Collection (ATCC 33277)), *F. nucleatum* (ATCC 25586) and *S. oralis* (German Collection of Microorganisms and Cell Cultures (DSMZ 20627)). Culture and handling of all three strains were done in an anaerobic incubator under an atmosphere containing 80% nitrogen, 10% carbon dioxide and 10% hydrogen. For *P. gingivalis* and *F. nucleatum*, agar plate precultures were inoculated from previously prepared glycerol stock aliquots. *P. gingivalis* was plated from stock on Fastidious Anaerobe Agar (Oxoid Limited, Basingstoke, Hampshire, UK) supplemented with 5% defibrinated sheep blood (Oxoid Limited), once a week. Every other day, liquid cultures were prepared by inoculating Schaedler medium (Oxoid Limited), supplemented with vitamin K (10 µg/ml, Carl Roth GmbH & CO. KG, Karlsruhe, Germany), with *P. gingivalis* scraped from this plate. *F. nucleatum* was plated on Fastidious Anaerobe Agar supplemented with 5% defibrinated sheep blood once a week. Fastidous Anaerobe Broth (Oxoid Limited), medium supplemented with 5% horse serum (USA origin, Sigma-Aldrich Chemie GmbH, Munich, Germany), was inoculated every day with *F. nucleatum* scraped from the agar plate and cells for oral inoculation were taken every day from the liquid overnight culture. Glycerol stock aliquots of *S. oralis* were used to inoculate Brain Heart Infusion medium (BHI; Oxoid, Wesel, Germany), supplemented with vitamin K (10 µg/ml). Bacteria suspensions for oral inoculation were taken every day from these liquid overnight cultures.

### Oral inoculation procedure

For all bacterial strains used for oral inoculation, optical density at 600 nm and corresponding number of colony forming units were previously determined from overnight cultures. For oral inoculation, 10^9^ cells per strain per animal were resuspended in carboxymethyl cellulose and administered in an inoculum of about 100 µl to each animal of group B. To apply the inoculum into the oral cavity of each of the animals, we used a lab pipette with regular filter tips (Research plus, 1000 µl, Eppendorf, Hamburg, Germany). Oral inoculation was performed this way on 5 days per week for six consecutive weeks.

### Clinical inspection and assignment of a mucosa index (MI)

At the end of the experiment, 49 days after the installation of the experimental abutments in intervention 3, peri-implant health was assessed macroscopically, with the animals in deep anaesthesia before dissection. Detailed notes on the health status of the peri-implant mucosa were taken by a dentist experienced in animal studies, who was blinded to sample identity. Based on these notes, a mucosa index between 0 and 3, based on the gingival index described by Löe [46], was assigned to all implantation sites at which the complete implant system was retained. The mucosa index was scored as follows: 0 = normal mucosa; 1 = mild inflammation—slight changes in colour, slight oedema, no bleeding; 2 = moderate inflammation -redness, oedema and glazing, bleeding on probing; 3 = Severe inflammation, marked redness and oedema, ulceration, tendency to spontaneous bleeding.

### Dissection

Using an overdose of anaesthetics, all animals were sacrificed directly after clinical inspection at the end of the experiment. Abutments and retaining screws were removed from the implants and either frozen immediately in liquid nitrogen or fixed in glutardialdehyde for later microscopic analysis.

### Biofilm quantification on retaining screws

After the experimental abutments and retaining screws were removed at the end of the experiments, six abutment and retaining screw combinations (unmodified titanium) from group A and eight from group B were immediately used for fluorescent staining of the attached biofilm. After fixation with 2.5% glutardialdehyde (Carl Roth GmbH, Karlsruhe, Germany) in PBS, SYTO®9 (from the LIVE/DEAD® BacLight™ Bacterial Viability Kit, Life Technologies, Darmstadt, Germany) was applied at a 1:1000 dilution in PBS. Subsequent microscopic examination and quantification of the complete volume of the attached biofilm was done using a confocal laser scanning microscope (Leica TCS SP8, Leica Microsystems, Mannheim, Germany). The SYTO®9 dye was excited at 488 nm and the emission was measured from 500 to 545 nm. Microscopic examination and quantification of the complete volume of the attached biofilm was done by a researcher blinded to sample identity. Prominent features of the geometry of the retaining screw were used to allow a reproducible scanning procedure. The surface marked in green in Fig. 4A was scanned at a tenfold magnification during microscopic examination with a z-step size of 3 µm. The biofilm volume attached to the titanium surface was later quantified by analyzing the obtained z-stacks with the software IMARIS (Version 8.4, release 2016, Oxford Instruments, Abington, UK).

### Sampling and polymerase chain reaction to verify polymicrobial infection

DNA was isolated from all abutments and retaining screws at the end of the experiment, using either the Fast DNA-Spin Kit for Soil (MP Biomedicals, Eschwege, Germany) for abutments and retaining screws, which were frozen immediately after dissection, or the QIAamp® DSP DNA FFPE Tissue kit (QIAGEN, Venlo, Netherlands) for abutments after staining and CLSM analysis. On these DNA samples, PCR for specific amplification of the 16S-rRNA gene sequence of *P. gingivalis*, of the gtfR gene sequence of *S. oralis*, which encodes the glucosyltransferase, and of the *rpoB* gene sequence *of F. nucleatum*, which encodes the β subunit of the bacterial RNA polymerase, was performed. PCR primer sequences and conditions are given in Table 1. The primers used for the *P. gingivalis* and *S. oralis* PCR were used as published before [47, 48]. The primers used for the *F. nucleatum* specific PCR were designed for this study.

**Table 1:** Bacterial species-specific primers used in PCR

Species	Primer sequence	Strand	Size (bp)
*Porphyromonas gingivalis*	5′ AGGCAGCTTGCCATACTGCG 3′	+	404
	5′ ACTGTTAGCAACTACCGATGT 3′	−	
*Fusobacterium nucleatum*	5′ GCCTCATGGCTCTAAGGGAG 3′	+	165
	5′ ACCCCTTTGTTTCCATGCCT 3′	−	
*Streptococcus oralis*	5′ TCCCGGTCAGCAAACTCCAGCC 3′	+	374
	5′ GCAACCTTTGGATTTGCAAC 3′	−	

### Statistical analysis

Normal distribution of the data was accessed using the Shapiro–Wilk test and the Kolmogorov–Smirnov test. As the results of MI assignment did not pass these tests we performed further statistical analysis of the results of this analysis applying the non-parametric Mann–Whitney-test. Data of the biofilm quantification were distributed normally and so we applied an unpaired-two tailed t test for statistical analysis. For both analyses, the significance level was set to α = 0.05 and the software GraphPad Prism (Version 8.4.2, GraphPad Prism Software Inc., La Jolla, USAreleased on April 8th 2020) was used.

## Results

### Three-step implantation procedure

In this study, a three-step implantation procedure was applied, similar to the original Brånemark protocol for installation of dental titanium implants in patients [49, 50].

Of the 27 rats included in the experiment, none was lost due to excessive weight loss. Four animals were lost over the course of the experiment: two of these died due to complications during anaesthesia, one was removed from the study after both implants were found missing during the experiment. A fourth animals had to be sacrificed due to a sudden decline in its physical condition during the experiment (see below).

In the first intervention (Fig. 2A) implants were successfully placed on both sides in 24 animals. Two animals died during intervention 1 due to complications in anaesthesia. In one animal implant placement was successful only on one side. Overall, 49 implants were placed in 25 animals for osseous integration during stage 1. 46 of these implants were covered successfully with a gingival flap to allow osseous integration as unperturbed by external influences as possible. For three implants (from three animals) covering was only partially achieved.

At the time of the second intervention, six weeks later, 46 implants were still in place. Ten of these implants were still fully submerged beneath the mucosa, while 36 were either only partially covered by the mucosa (13/36) or not covered at all. Three implants were lost in the course of stage 1 and gingiva covered the vacant implantation sites. In the second intervention, healing abutments were attached to all 46 remaining implants to allow transmucosal healing (Fig. 2B). The healed mucosa was reopened at the ten implantation sites where the mucosa fully covered the implant. As most of the implants were not fully covered anymore but the mucosa had already healed around the penetration site, only minor incisions or a slight mobilization of the mucosa were necessary before healing abutments could be attached. At sites where the mucosa had healed around the implant, it was left unperturbed during healing abutment attachment.

During the following healing period one animal had to be euthanized with both implants still in place, due to a poor state of health, which was unrelated to the performed treatment. Three more implants were lost in the course of stage 2 healing (two of them in one animal, which was removed from the study when the loss was detected). In the third intervention, the healing abutments were replaced by experimental abutments for later biofilm analysis (Fig. 2C). At the time of the third intervention, 41 implants were still in place. Two more were removed during the third intervention due to insufficient osseous integration. Overall, 39 implants were still in place and could be fitted with 22 unmodified titanium abutments and retaining screws and 17 abutments and retaining screws which featured a surface functionalization. A completely assembled implant with experimental abutment and retaining screw is shown in Fig. 2D.

At the end of the experiment, 39 implants were still in place, but 9 abutments and retaining screws were lost. The remaining 14 modified and 16 unmodified titanium abutments and retaining screws could be re-isolated in the final dissection. Of the sixteen unmodified abutments and retaining screws analysed in this study 8 were isolated from control group A animals and 8 from group B.

### Clinical inspection of implantation sides before dissection

**Fig. 3:**
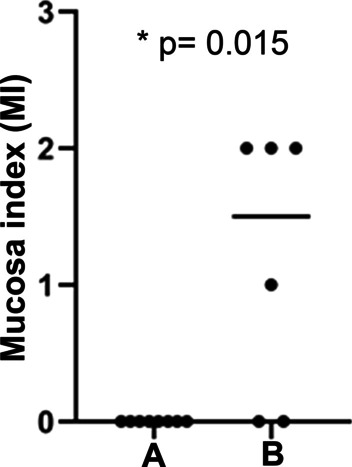
Clinical inspection of implantation sites at the end of the experiment using a mucosa index based on the gingival index introduced by Löe [46]. Criteria for the assignment of the mucosa index: 0 = normal mucosa; 1 = mild inflammation—slight changes in colour, slight oedema, no bleeding; 2 = moderate inflammation -redness, oedema and glazing, bleeding on probing; 3 = Severe inflammation, marked redness and oedema, ulceration, tendency to spontaneous bleeding. A = control group treated with antibiotics continuously (N = 8); B = group infected via oral inoculation with *P. gingivalis*, *F. nucleatum* and *S. oralis* (N = 6)

The outcome of the final clinical macroscopic inspection was described using a mucosa index. As the loss of the abutment might influence the health status of the peri-implant tissue, implants, which were no longer fully assembled, were not taken into consideration for the final assessment of the clinical situation in the two treatment groups. Overall, the mucosa index could be assigned to 8 implantation sites in animals from group A and 6 implantation sites from group B. The analysis revealed that 4 out of six animals from group B (experimental group) showed signs of mild to moderate mucosal inflammation (3 with score 2; 1 with score 1; 2 with score 0) whereas the peri-implant mucosa of all 8 control animals (group A) appeared healthy with no signs of inflammation (8 with score 0). This difference was statistically significant (*p* = 0.015) (Fig. 3).

### Biofilm on retaining screws

**Fig. 4:**
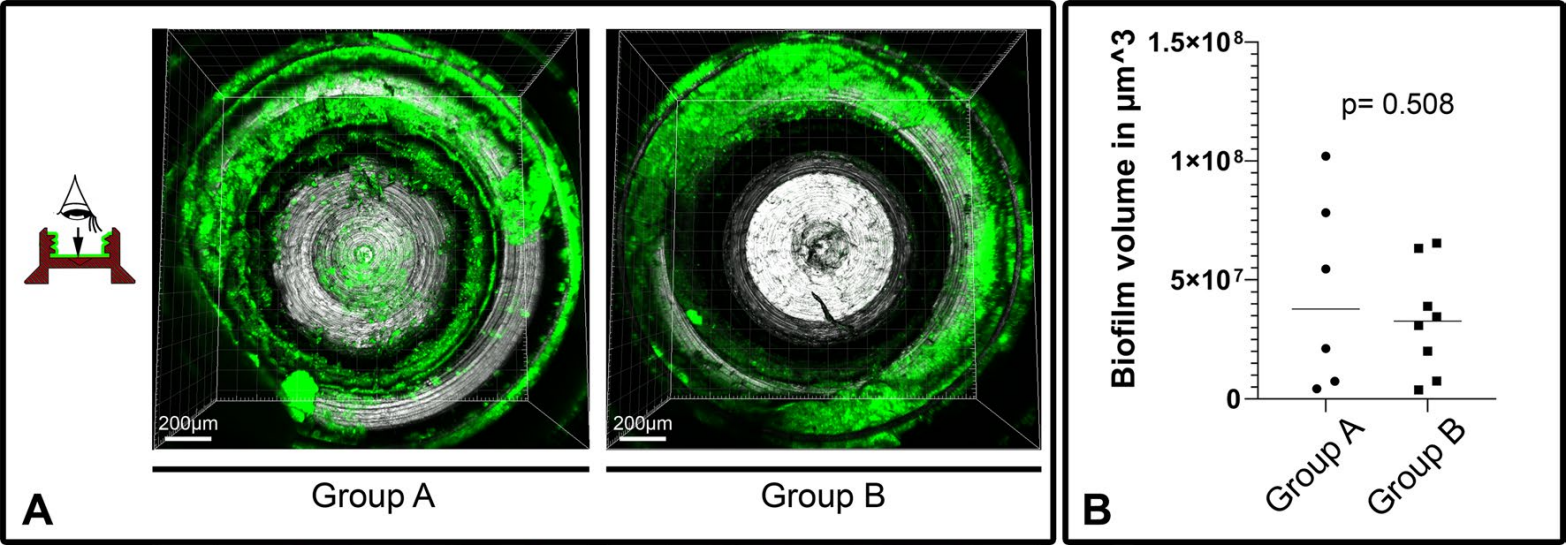
Quantification of biofilm on test surface inside unmodified titanium retaining screws. **A** Representative pictures of biofilm on uncoated titanium surface in group A and B detected via CLSM. **B** Statistical comparison of biofilm volume detected in the two treatment groups. A = control group treated with antibiotics continuously; B = group infected via oral inoculation with *P. gingivalis*, *F. nucleatum* and *S. oralis*. For group A N = 6, for group B N = 8

Extensive microscopic examination of the isolated abutments and retaining screws revealed that bacterial biofilms could most reliably be quantified on a defined surface area inside the retaining screws. Comparison of both treatment groups revealed no significant difference (*p* = 0.508) between animals treated with oral bacterial inoculation (group B) and animals continuously treated with ampicillin and kanamycin (group A) (Fig. 4). The results of the quantification also showed that the amount of biofilm was variable between the samples from different animals. It ranged between 4.28 × 10^6^ and 1.02 × 10^8^ µm^3^ in group A and between 3.83 × 10^6^ and 6.54 × 10^7^ µm^3^ in group B.

### Success rate of oral infection

To verify that the bacteria administered via oral inoculation had successfully established themselves as part of the biofilm on the titanium surfaces in vivo, DNA was isolated from all abutments and retaining screws re-isolated at the end of the experiment and subjected to a species-specific PCR analysis. *F. nucleatum* and *S. oralis* were detectable in all samples from group B and in none of the samples from group A. *P. gingivalis* was not detectable in either group (Table 2).

**Table 2:** Distribution of DNA samples with positive PCR results for the administered bacteria in both treatment groups

Treatment group	*P. gingivalis*	*F. nucleatum*	*S. oralis*
Group A	0/8	0/8	0/8
Group B	0/8	8/8	8/8

## Discussion

The aim of this study was to establish a novel rodent model for functional evaluation of new implant materials, which allows for a reliable quantification of bacterial biofilm on the implant material. For this purpose, we developed an experimental multi-part custom-made dental implant, consisting of an endosseous implant screw and a transmucosal abutment, and a protocol for a three-step implantation procedure in the diastema region of the rat maxilla. The implant design allows the removal of the experimental abutment together with the retaining screw at the end of the experiment, facilitating further analysis like microbiological and molecular examination of the attached biofilm, while preserving the endosseous part of the implant for other analyses. Using this procedure, three-dimensional biofilm formation could reliably be quantified by confocal laser-scanning microscopy. Oral inoculation with human-derived strains of *S. oralis*, *F. nucleatum* and *P. gingivalis* resulted in the integration of *S. oralis*, an important primary colonizer of human dental biofilm, and *F. nucleatum*, a “bridge organism” for further biofilm maturation and attachment of late colonizers, into the pre-existing oral flora of the rat and the implant associated biofilm. Animals with confirmed *S. oralis* and *F. nucleatum* integration in the oral biofilm showed signs of mild to moderate peri-implant mucositits, which was not detectable in control animals with permanent oral application of kanamycin and ampicillin.

## Novel three-step implantation procedure

The experimental implants designed for this study showed good osseous integration properties. In spite of the abutment exchange, eight weeks after endosseous implantation, which certainly meant a strain on the implant-bone interface and requires a certain stability of the implant, implantation was successful in 79.6% of the cases.

The optimal implantation site for an endosseus experimental implant in the oral cavity of the rat is still under discussion. Freire et al*.* reported a good primary stability of one-piece implants after placing them in the alveolar ridge of the diastema region in rats. After six weeks, one out of six implants had exfoliated in the control group of their experiment, yielding a success rate of 83.3% [51]. To our knowledge, Koutouzis et al*.* [41] described the only two-step installation of an experimental dental implant in the rat so far, using the healed extraction site of the first maxillary molar for implantation. Although they reported the loss of several animals due to surgery related issues and self-injury of the animals, the rate of implants with successful osseous initial integration in the remaining animals is at 78.6% remarkably similar to the one of this study. Based on the results of these studies and our own, we suggest that implantation in the diastema region as well as in the molar extraction site can result in a good primary stability of endosseous dental implants in the rat.

The design of the implant system and the implantation procedure applied in this study was based on the original Brånemark procedure described for dental implant patients [49]. Although the original two-step implantation procedure by Brånemark is partially replaced today by regimes of early or even immediate loading, too early loading is still considered a thread to long-time implant stability, if the primary stability is not perfect [52, 53]. Both in patients and in the rat model, osseous integration of implants can be impeded if the implant head protrudes into the oral cavity to the level of the occlusal surface of the molars, imitating the situation of too early loading. Even minute movements of the implant during osseous integration can interfere with the establishment of a direct bone-implant interface, resulting instead in an implant ensheathed by connective tissue [49]. The use of an implant which is assembled step by step, as in the present study and the publication by Koutouzis et al*.* [41], can probably help to prevent this harmful effect in experimental implantation.

A major focus of this study was to establish an implant system in the animal model, which allows an easy insertion and removal of the implant abutment. Our multi-part implant design and sequential implantation procedure enable the attachment of the experimental abutment to the implant right before the oral inoculation and biofilm colonization by the introduced bacteria. In this way, wear effects and abrasion or early colonization by endogenous bacteria can be avoided before the test period. Without prolonged exposure before the actual testing conditions are established, masking of potential antibiofilm effects is reduced, which facilitates the final evaluation of the tested material or modification. Also, experimental abutments and retaining screws can be removed separately from the osseous integrated part of the implant at the end of the experiment. This feature allows the final quantitative and qualitative analysis of the biofilm on experimental abutment and retaining screw, sparing the osseous integrated part of the implant for further analysis.

Concerning the first healing stage of the implantation procedure, a higher number of implants fully submerged under the mucosa would be desirable to further improve the comparability of the starting conditions between the individual samples. A disadvantage of the three-piece-design of our implant that has to be taken into account is the possible loss of experimental abutments. During the final seven weeks of our experiment, we documented a loss of nine out of thirty-nine abutments. The losses mainly occurred during the first week after abutment exchange (8/9).

## Effect of the bacterial infection

One of the aims of this project was the introduction of bacterial key-organisms of human dental biofilm into the microbial community present in the oral cavity of specific pathogen-free rats. To avoid false positive results in the final PCR-based detection of the inoculated bacteria in the implant-attached biofilm, bacterial inoculation was terminated one week before dissection. Thus, administered bacteria, which remained in their planktonic form or which had only temporarily attached to an oral surface, should have been eliminated from the oral cavity by mechanical stimuli and swallowing by the time of sampling. We were able to confirm the presence of *S. oralis* and *F. nucleatum* DNA in samples obtained from titanium abutments and retaining screws in all animals of experimental group B and in none of the control group animals via species-specific PCR. This indicates that *S. oralis* and *F. nucleatum* were successfully established in biofilms on the implant material.

Although streptococci have been found to be part of the endogenous oral microbiota of the rat with a relatively high abundance of about 20% [54], a regular oral inoculation with a human-derived strain of *S. oralis*, was sufficient to achieve its integration into the pre-existing oral microbiota and biofilm. *S. oralis* belongs to the yellow complex of plaque bacteria, which is commonly not associated with periodontal or peri-implant infections, but rather with a healthy state of periodontal tissues [25, 55, 56]. That is why it is usually not used in animal models for periodontal or peri-implant infections. Nevertheless, *S. oralis* is one of the most common species among the cluster of early colonizers, which pave the way for any oral biofilm [23]. For that reason, it will be particularly useful for implant material evaluation in this model. A surface modification which effectively reduces colonization by streptococci will most likely help to prevent all later steps of biofilm maturation as well. Fusobacteria are considered to be “… central structural components of plaque and essential for plaque maturation and an increase in plaque diversity…” [23]. *F. nucleatum* has recently been described to appear in dental implant associated biofilms at the time when the shift from a healthy to a pathogenic biofilm takes place and signs of peri-implant mucositis become apparent. For this reason, it is considered one of the most important members of a peri-implantitis related complex of oral bacteria [8].

At the end of our experiment, we observed clinical symptoms indicating peri-implant mucositis in four of the six animals in group B, with bleeding on probing occurring in three of these four animals, whereas none of the antibiotics-treated control group animals showed any signs of inflammation in the implant area. These findings indicate that *S. oralis* and *F. nucleatum*, in combination with the endogenous oral microbiota of the rats, were sufficient to induce a phenotype of mild to moderate peri-implant mucositis. Also, the lack of inflammation in the antibiotics-treated control group animals indicates that the reduction of bacterial biofilms, in the situation of a material test experiment via the application of a surface modification, will probably result in a reduction of clinical signs of inflammation, establishing a good experimental readout for material evaluation. Obviously, like any other animal model, this newly developed model of in vivo biofilm-formation on dental implants has its limitations in mimicking all the aspects of the situation in human patients. The third inoculated human microbiota-derived bacterium, *P. gingivalis*, was detectable neither in group B nor in group A samples. Potential reasons, such as differences in coaggregation efficiency between certain combinations of individual bacterial strains, will be examined in further studies. The lack of colonization with *P. gingivalis* might account for the rather mild to moderate clinical symptoms observed in the animals. To mimic the situation of advanced peri-implantitis in patients and elicit more severe symptoms, it would surely be necessary to inoculate the animals with a combination of pathogenic late colonizers. For the testing of new implant materials though a special focus should be on early colonization as this is the aspect of biofilm development which should primarily be prevented by these materials and modifications. These early steps of biofilm formation are reproduced well in our model, as the primary and intermediate colonizers *S. oralis* and *F. nucleatum* are obviously part of the implant associated biofilm.

## Biofilm development and quantification

The aim of this study was to establish an in vivo model to test implant materials for their biofilm reducing properties. To this end, a method for reliable and reproducible quantification of biofilm on the implant material surface was needed.

As elaborated in a preliminary experiment (data not shown), the amount of biofilm on the outer surface of the experimental abutments in both treatment groups was very variable. Two possible explanations for this variation are mechanical destruction of the attached biofilm during the removal of the abutments at the end of the experiment or disturbed biofilm development on this relatively exposed surface throughout the experiment due to constant mechanical manipulation in the oral cavity. In search of a suitable test surface which is reliably colonized by biofilm, we identified the hollow space within the retaining screw as an area which was colonized by bacteria invading this cavity from the outside, and which was relatively protected from mechanical disturbances during the experiment.

To quantify the biofilm in this area, we applied a modified version of a protocol developed in our group for biofilm quantification via confocal laser scanning microscopy on flat, regularly shaped test bodies out of non-transparent materials [57]. The major modification was in the adjustment of the procedure for an irregularly shaped surface. We solved this problem by using landmarks of the retaining screw geometry as reference points to determine a region for reproducible scanning and, thus, could reliably detect three-dimensional biofilm on the bottom and side walls inside the retaining screw. As our results showed, the amount of biofilm detected in this way is still very variable between samples. It will be recommendable to use a sufficiently great number of samples for the testing of new materials in this model and to set up the experiment for paired comparisons involving one unmodified and one modified sample within each animal.

No differences were detectable in the amount of biofilm between antibiotic-treated animals of group A and animals from group B. The fact that we found biofilm formation in control group animals despite the antibiotic treatment is not surprising. It was to be expected that ampicillin and kanamycin would not be sufficient to permanently suppress all bacterial growth, although the composition of the oral bacteria available for this colonization was probably different in these animals after weeks of antibiotic treatment. We identified the cavity under the retaining screw as an area of reliable biofilm formation, comparable to the well-known clinical situation of bacterial leakage from the inside of multi-component implant systems [30, 31]. For coatings or surface modifications intended for these challenging areas, which cannot be reached during routine oral hygiene procedures, this model will be of particular use.

## Conclusions

In this study, we developed a novel small animal model to analyze the biofilm formation on dental implant systems additionally including human-derived strains of *S. oralis* and *F. nucleatum*. The central structural function of both *S. oralis* and *F. nucleatum* in biofilm formation and maturation towards the pathogenic shift make the establishment of both species in an in vivo biofilm on experimental dental implants an achievement of some potential for further studies. The resulting model allows not only for in vivo evaluation of new implant materials but also for experimental addition of a variety of late colonizers, which are known to coaggregate with *F. nucleatum* and could be tested for synergistic function in the pathogenesis of peri-implantitis. Quantification of biofilms on dental implants in animal models via CLSM analysis, as described in this study, can be used in the future as a powerful tool for functional screenings of newly developed implant materials or material modifications.

## Data Availability

The datasets used and/or analyzed during the current study are available from the corresponding author on request.
